# Spatial reactant distribution in CO_2_ electrolysis: balancing CO_2_ utilization and faradaic efficiency†

**DOI:** 10.1039/d1se01534f

**Published:** 2021-10-27

**Authors:** Siddhartha Subramanian, Joost Middelkoop, Thomas Burdyny

**Affiliations:** Materials for Energy Conversion and Storage (MECS), Department of Chemical Engineering, Faculty of Applied Sciences, Delft University of Technology van der Maasweg 9 2629 HZ Delft The Netherlands T.E.Burdyny@tudelft.nl

## Abstract

The production of value added C1 and C2 compounds within CO_2_ electrolyzers has reached sufficient catalytic performance that system and process performance – such as CO_2_ utilization – have come more into consideration. Efforts to assess the limitations of CO_2_ conversion and crossover within electrochemical systems have been performed, providing valuable information to position CO_2_ electrolyzers within a larger process. Currently missing, however, is a clear elucidation of the inevitable trade-offs that exist between CO_2_ utilization and electrolyzer performance, specifically how the faradaic efficiency of a system varies with CO_2_ availability. Such information is needed to properly assess the viability of the technology. In this work, we provide a combined experimental and 3D modelling assessment of the trade-offs between CO_2_ utilization and selectivity at 200 mA cm^−2^ within a membrane-electrode assembly CO_2_ electrolyzer. Using varying inlet flow rates we demonstrate that the variation in spatial concentration of CO_2_ leads to spatial variations in faradaic efficiency that cannot be captured using common ‘black box’ measurement procedures. Specifically, losses of faradaic efficiency are observed to occur even at incomplete CO_2_ consumption (80%). Modelling of the gas channel and diffusion layers indicated that at least a portion of the H_2_ generated is considered as avoidable by proper flow field design and modification. The combined work allows for a spatially resolved interpretation of product selectivity occurring inside the reactor, providing the foundation for design rules in balancing CO_2_ utilization and device performance in both lab and scaled applications.

## Abstract 2

The production of value added C1 and C2 compounds within CO_2_ electrolyzers has reached sufficient catalytic performance that system and process performance – such as CO_2_ utilization – have come more into consideration.

## Graphical abstract

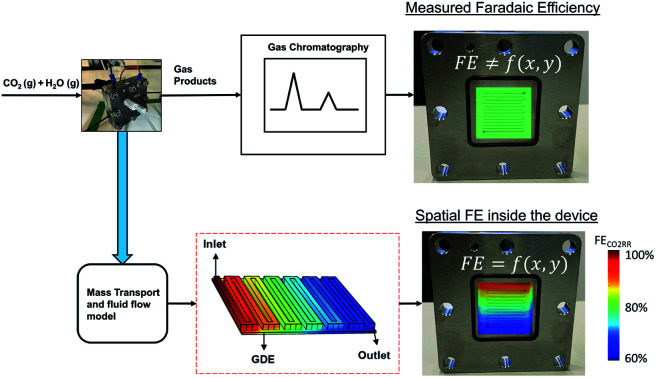

## Introduction

One of the emerging technologies to mitigate fossil fuel-based carbon emissions is the electrochemical conversion of CO_2_ to fuels and value-added products. In electrochemical CO_2_ reduction, an electric potential is applied in the presence of an appropriate catalyst to convert CO_2_ and H_2_O to syngas (CO + H_2_), ethylene (C_2_H_4_), ethanol (C_2_H_5_OH) and formate (HCOOH) among other products.^1–4^ To meaningfully mitigate CO_2_ emissions and be cost-competitive with alternative production routes, CO_2_ electrolyzers will need to be proven as scalable to global production rates on the order of 100's of Mtons per year.^5–7^ While water electrolyzers are developmentally able to reach such scales, CO_2_ electrolyzers are at a much earlier stage of development. Thus, while producing an anthropogenic carbon cycle composed of converting atmospheric CO_2_ to fuels using solar and other renewable energy sources is appealing, additional research and development is needed to improve the performance metrics and scale of the technology for it to become a viable option.^8–10^

To perform research into CO_2_ electrolyzers at increased production rates, a greater fraction of research has taken place under elevated current densities (>100 mA cm^−2^), using either high pressure systems or gas diffusion electrodes to enhance the availability of CO_2_ at the catalyst surface. Gas diffusion electrodes (GDE) in particular have been found to be promising due to their ease of operation at atmospheric conditions which lowers the barrier for research to adopt their use.^11–13^ When paired with novel catalyst architectures and cell designs, CO_2_ electrolysis on GDEs has then achieved current densities on the order of 1 A cm^−2^ for promising products such as both CO^14^ and ethylene^15^ with reasonable faradaic efficiencies and cell voltages. Additionally, some researchers have begun discussing the importance of CO_2_ utilization (as known as single-pass conversion efficiency) within such systems. Separate works have assessed the maximum conversion for a given configuration,^16^ the crossover of the CO_2_ to the anode as carbonate,^17^ and the observed drop in faradaic efficiency at higher CO_2_ utilizations.^18^ Such research has made it clear that trade-offs will ultimately exist between the traditional performance metrics of the CO_2_ electrolyzer itself (current density, faradaic efficiency, overpotential), and the efficiency and cost of the entire CO_2_ conversion process consisting of upstream and downstream processes.^19^

The balance between CO_2_ utilization and faradaic efficiency is particularly interesting as these metrics are directly impacted by the gas flow rate, the applied current density, temperature and the electrolyte alkalinity, all of which affect the CO_2_ that is available for conversion. For example, Jeng *et al.*^16^ highlighted the trade-off between partial current density for CO and the fraction of CO_2_ converted to products for a 25 cm^2^ membrane-electrode assembly (MEA) CO_2_ electrolyzer under various operating conditions, noting a consistent maximum CO_2_ utilization of 43% for the given reaction. While such observations provide valuable information around CO_2_ utilization in such systems, the trade-off in faradaic efficiency with CO_2_ utilization under varying CO_2_ concentrations has received less attention and is less well-described. Specifically, while the CO_2_RR faradaic efficiency of a system under excess flow conditions can be determined using either a high gas flow rate or a very small geometric surface area (*e.g.* <1 cm^2^), the selectivity of the system under decreasing CO_2_ partial pressures is less clear with only a few studies available.^20^ Importantly, as the surface area of standard test cells increases, *the concentration of CO*_*2*_*will also vary spatially throughout the reactor*, leading to spatial differences in reactivity and faradaic efficiency that will need to be understood to scale-up and optimize the technology.

While the influence of spatial reactant distribution on performance has not been well-investigated in the CO_2_ electrolysis community, there is a wealth of research in the fuel cell community assessing the influence of reactant concentrations, flow patterning and under-rib convection on efficiency, utilization and mass transport on the overall performance of the device.^21–24^ Using previous electrochemical fields as a guidepost, it is apparent that understanding the spatial variation of selectivity within a CO_2_ electrolyzer device will also be an essential step towards scaling-up such devices as well as choosing configurations which maximize CO_2_ utilization without unnecessary penalties in selectivity. For CO_2_ electrolysis, these efforts are complicated by competing and homogenous reactions which poses additional challenges as compared to well-studied parallel electrochemical fields. There is also less data presently available evaluating the performance differences between different flow fields for the gaseous CO_2_ channel as most research is performed using smaller geometric catalyst areas and a fully open cavity.

**Fig. 1:**
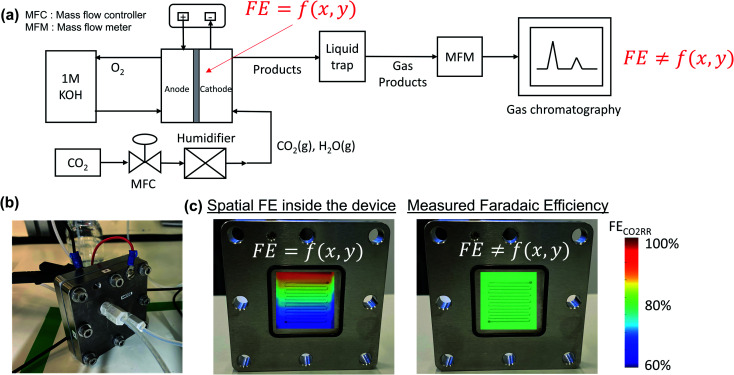
(a) Simplified schematic of the experimental setup used for CO_2_ electroreduction to CO in a membrane electrode assembly (MEA). (b) Figure of the experimental MEA utilized in the work. (c) Overlaid schematic of the actual *vs.* measured faradaic efficiency of a CO_2_ electrolysis system under CO_2_-limited operating flow rates for the serpentine flow fields used for CO_2_ flow behind a gas-diffusion layer.

Here, we sought to provide a framework for how reactant flow rate and spatial CO_2_ distribution impacts product selectivity at higher CO_2_ utilizations using a well-utilized electrochemical testing platform. Firstly, we performed CO_2_ electrolysis using a silver (Ag) gas diffusion electrode in a 5 cm^2^ MEA at various reactant flow rates to determine the macroscopic influence on product selectivity. From these experiments a ‘black box’ evaluation of faradaic efficiencies (FE) at various CO_2_ utilizations is defined. We then built a 3D mass transport model of the cathode side of the MEA to estimate the spatial CO_2_ distribution inside the reactor and catalyst layer under each of the varying flow conditions to convert the ‘black box’ results of the CO_2_ distribution throughout the 5 cm^2^ cell into a more spatially resolved interpretation of reactant concentration at the catalyst's surface (Fig. 1). Finally, we show that by using a combined experimental and modelling approach, the influence of reactant flow rate and spatial CO_2_ distribution can in turn be used to predict a spatial product selectivity across the device. Once defined, such a combined experimental and modelling system can then be used to predict the impacts of varying flow fields, cell areas and current densities, providing the groundwork for designing and prototyping CO_2_ electrolyzers which balance CO_2_ utilization with product selectivity.

## Results and discussion

Product quantification within gaseous-fed CO_2_ electrolyzers is presently performed by measuring the composition of the outlet gas phase using a gas chromatography (GC), and measuring the composition of the liquid electrolyte phases using nuclear magnetic resonance spectroscopy (NMR) or high performance liquid chromatography (HPLC). Such measurements provide a point-in-time ‘black box’ interpretation of the FE at a given flow rate, current density and configuration that can be monitored through periodic measurements (Fig. 1a). At elevated inlet flow rates where CO_2_ utilizations are low, the outlet gas stream remains >90% CO_2_ and it is subsequently assumed that ample CO_2_ can reach the entire catalytic surface area. In other words, no specific area of the catalyst surface exhibits mass transport limitations and the faradaic efficiency is assumed to be equal across the entire catalyst area (*e.g.* FE ≠ *f*(*x*,*y*)). Such an assumption is particularly valid for smaller catalyst areas, high CO_2_ flow rates and open cavity gas channels which are assumed as well-mixed and maintained at similar temperature and pressures.

As industrial and lab geometric cell areas increase, CO_2_ must be distributed to the GDL and catalyst area through flow fields, which are also critically acting as a current collector to ensure homogenous electrode potentials. Within these CO_2_ flow channels, the reactant and product compositions will then change along the length of each flow channel^25^ as the catalyst consumes CO_2_ and produces products such as CO and H_2_. In cases where CO_2_ utilizations are increased, spatial variations in performance and selectivity will occur when areas of the catalyst no longer have access to sufficient CO_2_, and produce unwanted H_2_ instead (see Fig. 1b for representation).^26–28^ To begin assessing this trade-off we first collected a data set under varying flow rate conditions for CO_2_ conversion to carbon-monoxide (CO) on a silver (Ag) catalyst in a membrane-electrode assembly with a serpentine flow field of 5 cm^2^ geometric area (Fig. S2†).

**Fig. 2:**
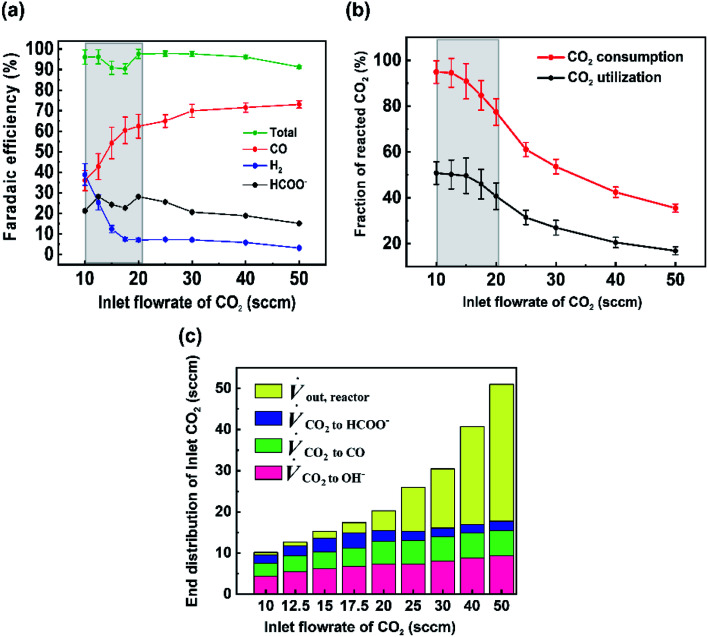
(a) Faradaic efficiency of products for various inlet flow rates performed at a current density of 200 mA cm^−2^. (b) CO_2_ utilization and CO_2_ consumption for different inlet flow rates at 200 mA cm^−2^. Greyed regions represent trade-offs between utilization and selectivity. (c) Carbon balance on cathode showing the volumetric flow rate of CO_2_ consumed to different reactions. Error bars represent the systematic error of the mass flow meter.

For the data set we performed electrolysis at a constant current density of 200 mA cm^−2^ for 3600 seconds at inlet CO_2_ flow rates between 10 and 50 sccm. The gas products and unreacted CO_2_ were quantified using a mass-flow metre (MFM) and GC installed at the exit of the reactor (Fig. 1a). As shown in Fig. 2a, we found that at excess flow rates between 20 and 50 sccm the faradaic efficiency of CO_2_ reduction products (CO and formate) was maintained between 93–97%, indicating that sufficient reactant is available throughout the system. At lower flow rates (<20 sccm), however the FE of hydrogen begins increasing steadily with increasing CO_2_ utilization, reaching an H_2_ selectivity of 38.9% at 10 sccm and a measured CO_2_ utilization of ∼50% (Fig. 2b). Over the entire examined region, CO_2_ utilization decreases with an increase in the inlet flow rate from 50.8% at 10 sccm to 16.8% at 50 sccm as shown in Fig. 2b. The highlighted grey region in Fig. 2a and b represents the likely operating region of a commercial CO_2_ electrolyzer as it best balances selectivity and utilization. Understanding and quantifying the performance trade-off is necessary to manufacture performance curves for CO_2_ electrolyzers, similar to other applications where trade-offs exist (*e.g.* centrifugal pumps). Such data is essential for positioning CO_2_ electrolyzers within integrated process and cost models that assess a broad operational parameter space. Additionally, better design of the reactant flow fields and gas-diffusion layers may improve performance further.

To better quantify the trade-off in utilization and selectivity, the available CO_2_ for reduction in the system must be known. To track this a carbon balance of the system is performed at various flow rates (Fig. 2c). In this analysis the inlet and outlet flow rates of CO_2_, CO and formate are all measured directly, with the exception of CO_2_ crossing the membrane as carbonate ions which was assumed to complete the carbon balance. Observing the trends in carbon flow rates, two interesting points arise. First, even under low flow rates of 10 sccm, some CO_2_ is observed in the outlet of the reactor (∼5%/v) even though the reaction appears CO_2_-limited. This indicates a measure of transport limitations between the serpentine gas channel and the catalyst's surface as a result of transport through the gas-diffusion media and into the catalyst layer. And second, the consumption of CO_2_ by OH^−^ ions is non-linear and varies with the availability of CO_2_ throughout the reactor. Both of these observations can be qualitatively interpreted from the presented data, but lack a quantitative interpretation in their present form as a result of the ‘black box’ measurement approach. Thus, a numerical transport model built upon the experimental results can be used to provide further understanding.

### Modelling CO_2_ spatial distribution

**Fig. 3:**
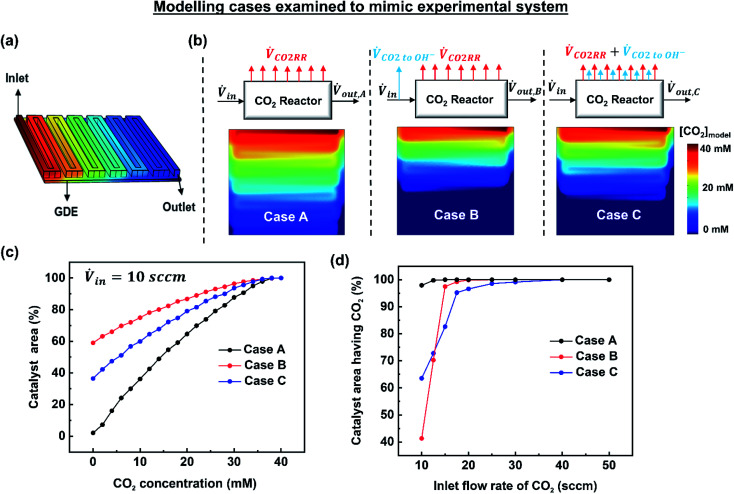
(a) 3D model of the flow channel and gas diffusion electrode. (b) Modelling cases examined to mimic the experimental observations. Shown here are the simulation results of CO_2_ concentration at the catalyst surface for an inlet flow rate of 10 sccm and 200 mA cm^−2^, (c) a cumulative distribution plot for the three cases showing the [CO_2_] distribution at the catalyst surface, (d) portion of catalyst surface having access to CO_2_ ([CO_2_] > 0) for all the inlet flow rates studied experimentally.

To gain deeper understanding of the reactant distribution inside the reactor, a 3D model of the mass transport and fluid flow in the cathode compartment of the MEA cell was created using COMSOL Multiphysics (Fig. 3a). The ultimate goal of the model is to provide a simple estimate of the concentration of CO_2_ at the surface of the catalyst layer for various operating conditions, which can then be used to predict a spatial and average faradaic efficiency (FE = *f*(*x*,*y*) and FE_average_). The predicted average FE of the system in particular provides a comparison to the experimental data, while the spatial assessment is useful to advance performance further and for the design of scaled systems beyond 5 cm^2^.

Included within the model are the CO_2_ serpentine gas channel and a gas-diffusion layer composed of a carbon fibre backing and a microporous layer (Fig. S6†). The gas-diffusion electrode is then modelled as a porous media similar to other works.^29^ In the model an inlet flux of CO_2_ is provided to the system in the gas channel, while a fixed current density is imposed at the surface of the gas-diffusion electrode to model the electrochemical reactions and consumption of CO_2_ by the electrolyte. The physical parameters and properties used in the model are shown in Table S6.†

Due to the complexity of constructing a fully-representative macroscopic and nanoscopic transport model, we have chosen to set our system boundaries at the interface of the microporous layer and the catalyst layer. The model then does not directly take into account the interaction between the catalyst layer and the membrane, 3D transport effects within the nanopores of the catalyst layer, or the homogenous CO_2_/HCO_3_^−^/CO_3_^2−^ reactions occurring within the liquid water and Sustainion membrane. To account for this we have constructed three modelling scenarios using experimental mass flows as inputs to construct different empirical models that highlight the effect of different scenarios on CO_2_ distribution. The most representative system is then used to continue the discussion on CO_2_ utilization and faradaic efficiency.

The three examined cases are as follows: in Case A, we ignore the fraction of CO_2_ reacting with hydroxide ions. In Case B, the amount of CO_2_ lost to hydroxide ions is subtracted at the inlet resulting in a reduced inlet flow rate. In Case C, the fraction of CO_2_ lost to hydroxide ions is assumed to occur homogenously throughout the catalyst surface. These three cases are visually depicted in Fig. 3b along with their resulting simulated CO_2_ concentrations at the catalyst layer interface at 10 sccm and 200 mA cm^−2^. Fig. 3c shows the analysed data set from Fig. 3b represented as a cumulative distribution function for the percentage of the catalyst area with a minimum concentration of CO_2_.

#### Case A: Modelling the cathode without accounting for CO_2_ reacting with OH^−^ ions

In this approach, CO_2_ losses due to its reaction with OH^−^ ions forming bicarbonate and carbonate ions are ignored. The results obtained for an inlet flow rate of 10 sccm at 200 mA cm^−2^ are shown in Fig. 3c, where the two-dimensional data set has been converted into a cumulative distribution functions as a percentage of the geometric area of the catalyst layer. Thus the percentage of catalyst area with ample and deficient CO_2_ can be visualized (Fig. S8†). From Fig. 3b it can be seen that the CO_2_ concentration decreases from the inlet to the outlet of the gas channels and at the catalyst surface. As shown in Fig. 3b, the cumulative distribution plot for CO_2_ at the catalyst surface shows that only 2.1% of the catalyst area is deficit of CO_2_ for an inlet flow rate of 10 sccm. Hence, Case A shows almost no CO_2_ limitation indicating that this reactant feed is sufficient to sustain the current density that is applied (200 mA cm^−2^). However, as could be expected, Case A clashes with the experimental observation of a low CO selectivity (35.9%) and a relatively high H_2_ selectivity (38.9%) at 10 sccm. This discrepancy between the modelling and the experimental results shows that CO_2_ losses (due to its reaction with OH^−^) cannot be ignored in modelling the spatial CO_2_ distribution.

#### Case B: Modified inlet flow rate approach

In Case B the inlet boundary condition of CO_2_ flux has been reduced to account for the amount of CO_2_ lost to OH^−^ ions over the entire reactor. Here, the amount of CO_2_ lost to OH^−^ ions was experimentally measured and subtracted from the inlet flow rate to obtain a modified inlet flow rate (Table S2†). In contrast to Case A, using the modified inlet flow rate approach a significant portion of catalyst surface (59%) is deficit of CO_2_ at 10 sccm (Fig. 3b). Although this agrees with the experimental observation of an increased H_2_ production (38.9%) at low flow rates, the change in catalyst area with access to CO_2_ is too abrupt under varied flow rates (Fig. 3d), which does not pair well with the gradual change in selectivity seen in the experiments (Fig. 2a). The flaw in a modified inlet flow rate approach is that the CO_2_ losses to OH^−^ ions are not distributed throughout the catalyst surface, meaning that the CO_2_ available in the front half of the serpentine channel is unfairly limited. Case B is then too much of a simplification to predict the spatial CO_2_ distribution and device selectivity accurately.

Of note, using a modified inlet flow rate would also slightly impact the fluid velocity and pressure drop between the inlet and outlet, altering the actual physical phenomena occurring inside the reactor. Such an approach would then have significant effects when large flow rates are used where a significant pressure drop might exist between the inlet and outlet of the reactor. Critically, Case B over penalizes the CO_2_ concentration throughout the majority of the reactor as CO_2_ lost to OH^−^ ions near the exit of the reactor has been removed prior to the reactor inlet.

#### Case C: Modified current density approach (modified CO_2_ flux to the catalyst)

With the aim of predicting the 2D spatial CO_2_ concentration in the reactor while maintaining a simplified modelling approach, Case C aimed to spatially account for CO_2_ loss to OH^−^ as well. To institute this within the model without implementing pore scale phenomena and homogeneous reactions, we instead imposed a penalty current density (*j*_loss_) that accounts for the additional consumption of CO_2_. The magnitude of the imposed penalty current density was calculated using the experimentally-measured loss of CO_2_ at each independent flow rate (eqn (1) and Fig. 2c), resulting in an empirical representation of the experiment. This modified current density was then added to the actual applied current density term to provide the spatial rate of CO_2_ consumption (eqn (2)). Fig. S3† shows the modified current densities which have been imposed in the model as a result of Case C, with all current density above 200 mA cm^−2^ being deployed as a non-faradaic consumption of CO_2_.1
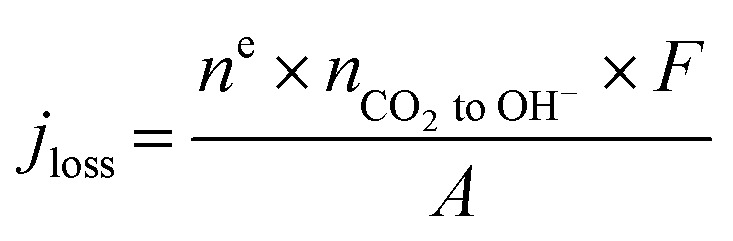
2
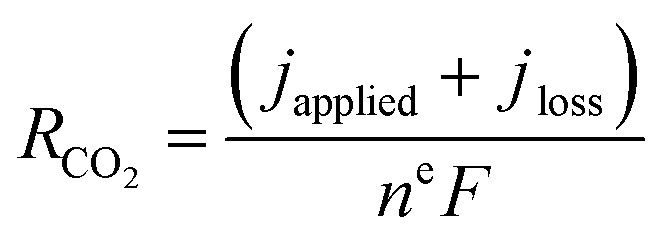
Here, *R*_CO_2__ is the reaction rate of CO_2_, *j*_loss_ is the modified current density calculated based on the amount of CO_2_ lost to OH^−^ ions (from experimental data), *n*^e^ is the number of electrons (2 for CO_2_ RR), *n*_CO_2_ to OH^−^_ is the moles of CO_2_ lost to OH^−^, *F* – Faraday's constant and *A* is the area of the catalyst surface (6.25 cm^2^).

Once imposed, Case C provides the spatial distribution of CO_2_ observed in Fig. 3b for an inlet flow rate of 10 sccm. Translating this to the cumulative distribution function in Fig. 3c, the net catalyst area with no access to CO_2_ is approximately 37%. Further, Fig. 3d shows the percentage of catalyst area with access to reagent results for all of the simulated cases and flow rates. Notably at flow rates within the utilization area of interest (10–20 sccm), Case C falls in between Cases A and B. The effect of parasitic CO_2_ loss is still not eliminated above 20 sccm, however, which can be attributed to poor CO_2_ access on the fringes of the gas-diffusion layer. In this case, this is due to the area of the GDE (6.25 cm^2^) expanding beyond the edge of the serpentine flow channel (5 cm^2^). Due to accounting for spatial effects, Case C is chosen as the most representative model for the remainder of the work.

### Predicted spatial and average faradaic efficiency

The previous section provided a set of models to predict the spatial concentration of CO_2_ within an experimentally-tested membrane-electrode assembly reactor. As the primary focus is to better understand the trade-offs between selectivity and utilization in these systems, these predicted concentrations of CO_2_ must be translated to a predicted spatial and average faradaic efficiency. To accomplish this we imposed the following selectivity criteria in eqn (3) and (4) based upon the predicted CO_2_ concentration, and the experimentally-measured faradaic efficiency under an excess CO_2_ flow rate of 50 sccm (97% CO_2_RR/3% HER). The data has been normalized to 100% (96.8% CO_2_RR/3.2% HER) for the purposes of the model.3
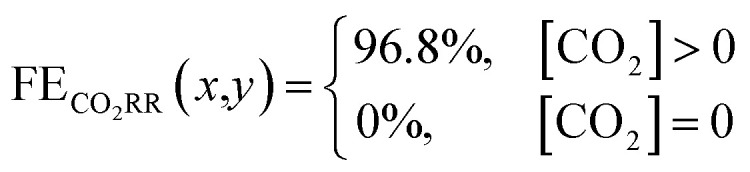
4
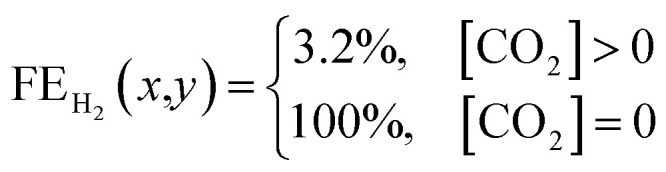


**Fig. 4:**
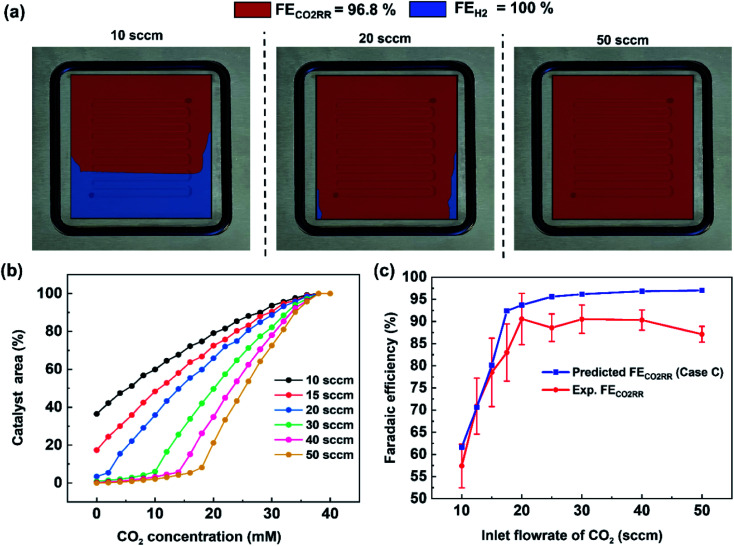
(a) CO_2_ concentration map at the catalyst surface determined from the numerical simulations showing the spatial CO_2_ distribution at various inlet flow rates, (b) a cumulative distribution plot of CO_2_ concentration at the catalyst surface for different inlet CO_2_ flow rates studied using a modified current density approach and (c) comparison of predicted faradaic efficiency of CO_2_RR with experimentally determined faradaic efficiency (FE_CO_ + FE_HCOO^−^_).

Using this criteria, the spatial faradaic efficiency across the catalyst layer of the GDE is visually shown in Fig. 4a for three different flow rates. Observing the low flow rate case of 10 sccm, the loss of selectivity towards CO_2_RR is shown to be primarily due to insufficient CO_2_ along the length of the reactor towards the outlet. In the 20 sccm case, however, it is only the edges near the outlet of the reactor that are expected to primarily produce H_2_ instead of CO_2_RR products. The plots in Fig. 4a for spatial selectivity are predicated on the assumption that there is not a transition region of selectivity between the shown blue and red regions. In an actual system the switch in selectivity from primarily CO_2_RR to H_2_ along the reactor of CO_2_-deficient system would be more gradual, but high selectivities are known to be possible even at lower partial pressures.^30^ A secondary check of the approach is to translate the spatially-predicted faradaic efficiency into a device-averaged FE like that reported experimentally.

The device-averaged FE can be calculated by using the distribution function in Fig. 4b for a variety of different flow rates, and combining this with the criteria presented in eqn (1) and (2). The resulting predicted FE of CO_2_RR and H_2_ for all the inlet flow rates studied are then shown in Fig. 4c, with the experimentally-measured values overlaid. It can be seen clearly that the predicted FE is in close agreement with the experimental FE of CO_2_RR, showing the promise for using predicted CO_2_ distribution within the reactor to predict spatial and average device selectivity. The consistent over prediction can be attributed to the experimental FE's being less than 100%, most likely due to the inability to capture all produced formate in MEA cell. Importantly both the trend in selectivity within the higher CO_2_ utilization region (10 to 20 sccm), as well as in the lower utilization range (20–50 sccm), follow the experimental data set well. Such a model forms the foundation for comparing GDE's with different permeability, flow fields with different geometries, and the trade-offs with selectivity and utilization under different current densities.

The model can also be used to draw new observations from the experimental data set. For example, the incremental change in CO_2_RR from 20–50 sccm is shown to be due to a CO_2_ deficiency on the outer edges of the domain where the larger gas-diffusion layer (6.25 cm^2^) loses access to CO_2_ from the 5 cm^2^ serpentine channel area (see 20 sccm plot in Fig. 4a). Such an area then only produced H_2_, which slightly lowers the “black box” measured FE *via* gas chromatography. We are then able to predict the location on the catalyst surface where CO_2_ limitation occurs, which can help in understanding and designing flow channel designs at the cathode.

Finally, we emphasize here that at an applied current density of 200 mA cm^−2^, there is an increase in the amount of CO_2_ reacting with OH^−^ ions with an increase in the reactant flow rate, which is identified in the increase in the *j*_loss_ value (Table S2†). This is quite reasonable since the local OH^−^ ions generated at 200 mA cm^−2^ is a constant (1.3 × 10^−5^ mol s^−1^) and an increase in the local CO_2_ concentration due to increased inlet flow rate shifts the reaction to the right producing more HCO_3_^−^ and CO_3_^2−^ ions. Moreover, this reduction in local [OH^−^] with increasing inlet flow rates would also reduce the local pH altering the reaction environment around the catalyst surface. A further increase in inlet flow rate (60–100 sccm) would result in the consumption of all the OH^−^ ions generated at the catalyst producing more HCO_3_^−^ and CO_3_^2−^ ions with a subsequent alteration of the local reaction environment. Operating at such high reactant flow rates would however reduce the CO_2_ utilization to less than 10% and also increase the pressure drop between the inlet and outlet (serpentine channel) resulting in an increased pumping power.^31^ Hence, optimizing the reactant flow rate to overcome CO_2_ mass transport losses as well as ensuring a high CO_2_ utilization and a low pressure drop is a challenge. Therefore, we restricted our focus of this study to flow rates of up to 50 sccm.

### Formate production from Ag GDE

**Fig. 5:**
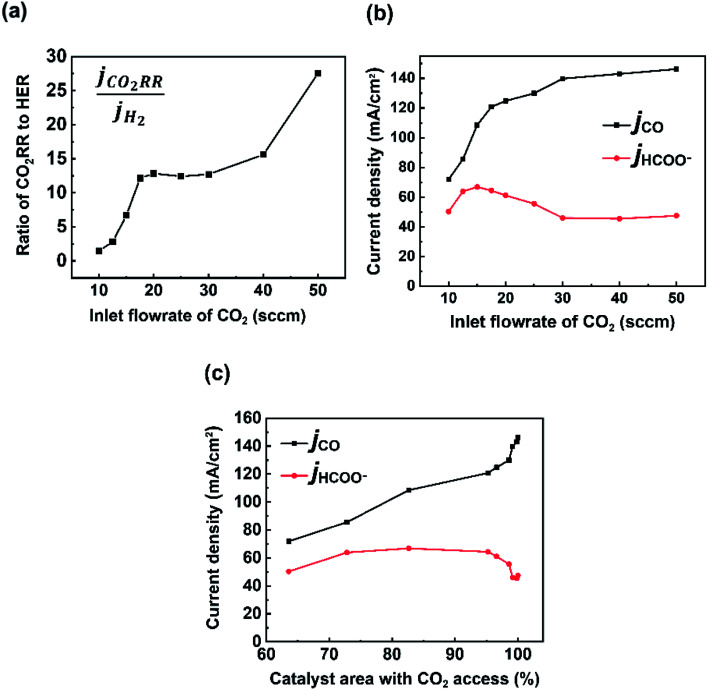
(a) Ratio of partial current densities of CO_2_ RR (CO + HCOO^−^) and H_2_. Partial current densities of CO and formate with (b) varying inlet CO_2_ flow rates and (c) catalyst area with CO_2_ access.

While much of the work here focused on the availability of CO_2_ and the subsequent CO_2_RR selectivity as a result of this, the experimental data set noted interesting and opposing trends in CO and formate selectivity under a variety of flow rate conditions (Fig. 2a). In particular while overall CO_2_RR *versus* HER trended downward as flow rates decreased as could be expected (Fig. 5a), the selectivity of CO to formate also followed a similar linear trend, both within the CO_2_-limited and non-limited flow rate regions (Fig. 5b). Here, we briefly contextualize these results and offer possible explanations given previous literature reports and our spatial model constructed here. It is worth noting that to measure formate we performed HPLC measurements of the anolyte samples post electrolysis for our Ag GDE system, meaning that only formed formate which crossed the anion exchange membrane could be measured, likely explaining some missing FE in our data set. We will provide speculation in spite of this.

The trend in CO to formate within the two flow rate regions have two possible explanations from literature: (i) the reaction pathway to formate exists through surface-adsorbed protons and competition with HER, (ii) formate selectivity supplants some CO selectivity under higher alkalinity conditions. The first point has been reported previously by Bohra *et al.*^32^ using DFT calculations which showed that *OCHO towards formate forms through a bound *H, whereas CO formation proceeds first through direct CO_2_ absorption. Thus, formate formation requires the Volmer step from HER formation in order to be formed. It would then be expected to see a lower CO/formate ratio when *H is more common, which would be the case in decreased and depleted CO_2_ conditions like those observed from 10 to 20 sccm. Regarding (ii), previous studies on GDE flow cells have shown increased formate/CO ratios under extremely alkaline conditions (11 M KOH in Seifitokaldani *et al.*^33^) and decreased formate/CO ratios under higher CO_2_ pressures (Gabardo *et al.*^34^). Both reports indicate that the pH of the reaction environment will influence the ratio of CO to formate produced. Within our system, this hypothesis could help to explain the decreasing trend in formate production as the inlet flow rate ranges from 20–50 sccm. At higher flow rates excess CO_2_ is available to negate the formed OH^−^ from the fixed current density reaction (see *V*_CO_2_ to OH^−^_ blocks in Fig. 2c). It is then likely that the reaction environment surrounding the catalyst layer leans to lower alkalinities at 50 sccm *versus* that of 20 sccm, even though ample CO_2_ is available in both cases. The experimental decrease in *j*_HCOO^−^_ is also seen when the model and experiments are combined (Fig. 5c), where formate current density drops when the full catalyst area has access to CO_2_.

### Operating feed rate for larger cells at high current densities

Within this study a serpentine flow channel was utilized to provide CO_2_ to a 6.25 cm^2^ catalyst area. The experimental and modelling results can be extended to reactor areas of various sizes and current densities, presuming the dominating phenomena are not altered by doing so. Here we first provide a calculation for the required flow rate of CO_2_ to balance utilization while maintaining higher CO_2_ reduction selectivity. We then comment on the important phenomena to consider in scale-up regarding CO_2_ feed rate.

To formulate the operating CO_2_ feed which best balances CO_2_ utilization and device selectivity for a given electrode area and current density, we utilized our 20 sccm flow rate as a base case. From Fig. 4a, the 20 sccm case best balances CO_2_ utilization (40%) and CO selectivity. Normalizing this flow rate with the geometric surface area of the GDE (6.25 cm^2^) and partial current density of CO (125 mA cm^−2^), we predict that the operating reactant feed for industrial operation should be 0.0256 cm^3^ min^−1^ mA^−1^. We compared this value with a study from Endrődi *et al.*^14^ where a similar study using Ag GDE in a zero gap CO_2_ electrolyzer at 1 A cm^−2^ was performed. In their study, a large geometric surface area of 100 cm^2^ was employed and a feed rate of 12.5 cm^3^ min^−1^ cm^−2^ was used to obtain the same CO_2_ utilization of 40%. Normalizing this feed rate to their CO partial current density (630 mA cm^−2^), the operating feed comes to 0.0198 cm^3^ min^−1^ mA^−1^ which agrees closely with our predicted value.

From the modelling studies performed we can comment that a number of factors would change the flow rate of CO_2_ required at the reactor inlet. Specifically, while the inlet flow rate can be well controlled, diffusion of CO_2_ from a gas channel into the liquid immersed catalyst layer is less tuneable and will be impacted by such things as specific device configuration, the catalyst thickness and deposition type, pressure drop within the system, temperature, *etc.* As reactors scale to larger and larger sizes these factors may alter the ideal CO_2_ feed rate. For example, for large reactors larger pressure drops in the gas-phase will occur if singular serpentine channels are used as the gas may prefer to shortcut under the gas channel and through the gas-diffusion layer. This would then result in a higher degree of under-rib convection changing localized CO_2_ concentrations from the 6.25 cm^2^ reactor. Thus, when moving from smaller to larger reactors, proper engineering design is needed to ensure that local phenomena are maintained near their ideal conditions, even as the reactor scales up.

## Conclusion

The balance between CO_2_ utilization and selectivity with electrochemical systems will be ever more important as CO_2_ electrolyzers are scaled to larger areas and considered within larger chemical processes due to implications they have on reliability, separation processes and system costs. The trade-offs in these metrics are currently measured and reported for an entire reactor, while being driven by spatial variation in concentrations across an entire electrochemical reactor. At present, the experimental ability for direct localized measurement of CO_2_ electrolysis products has not been demonstrated however. The work presented here aims to predict this trade-off by pairing bulk product measurement with a transport model to provide a measure of spatial resolution to our electrochemical cell. We believe that our approach can provide a starting point for a more extensive modelling study to enhance the understanding of the local reaction environment around the catalyst surface in a membrane electrode assembly configuration, employing anion exchange membranes. Importantly, we hope this work inspires experts from adjacent fuel cell community to provide their wealth of experience to accelerate the CO_2_ reduction field forward.

## Author contributions

S. S. completed all of the experiments and modelling work. J. M. setup the experimental apparatus and assisted with experimental issues. T. B. and S. S. conceived the project. All authors contributed to writing and editing of the manuscript.

## Conflicts of interest

There are no conflicts to declare.

## Supplementary Material

SE-005-D1SE01534F-s001
