# Supplementary material for: Spatial reactant distribution in CO2 electrolysis: balancing CO2 utilization and faradaic efficiency
Source: Sustain Energy Fuels. 2021 Oct 27;5(23):6040–8. doi: 10.1039/d1se01534f (PMC8610086; doi:10.1039/d1se01534f)
Supplement: SE-005-D1SE01534F-s001 [file SE-005-D1SE01534F-s001.pdf]

Electronic Supplementary Material (ESI) for Sustainable Energy & Fuels.
This journal is © The Royal Society of Chemistry 2021

Supplementary Information

Spatial reactant distribution in CO_2 electrolysis: Balancing CO_2 utilization and Faradaic Efficiency

Siddhartha Subramanian, Joost Middelkoop and Thomas Burdyny*

Corresponding author email: T.E.Burdyny@tudelft.nl

Materials for Energy Conversion and Storage (MECS), Department of Chemical Engineering, Faculty of Applied Sciences, Delft University of Technology, van der Maasweg 9, 2629 HZ Delft, The Netherlands.

Experimental

All experiments were performed in a 5 cm^2 area membrane electrode assembly (Dioxide materials) having a serpentine flow channel on both the anode and cathode endplates. Sigracet 38 BC gas diffusion layers (GDL) of 6.25 cm^2 area (2.5cm x 2.5cm) was used as the porous transport layer. Ag catalyst layer was deposited on top of microporous layer of GDL by direct current magnetron sputtering to obtain a thickness of 100 nm. Nickel foam (3 cm x 3 cm) was used as the anode. Ag GDE and Ni foam were combined with an oversized 16 cm^2 (4cm x 4cm) Sustainion anion exchange membrane (X37-50 Grade RT) to assemble the MEA (Fig. S2). An exchange MEA configuration using 1M KOH as the anolyte and humidified CO_2 as reactant at the cathode were fed into the reactor.

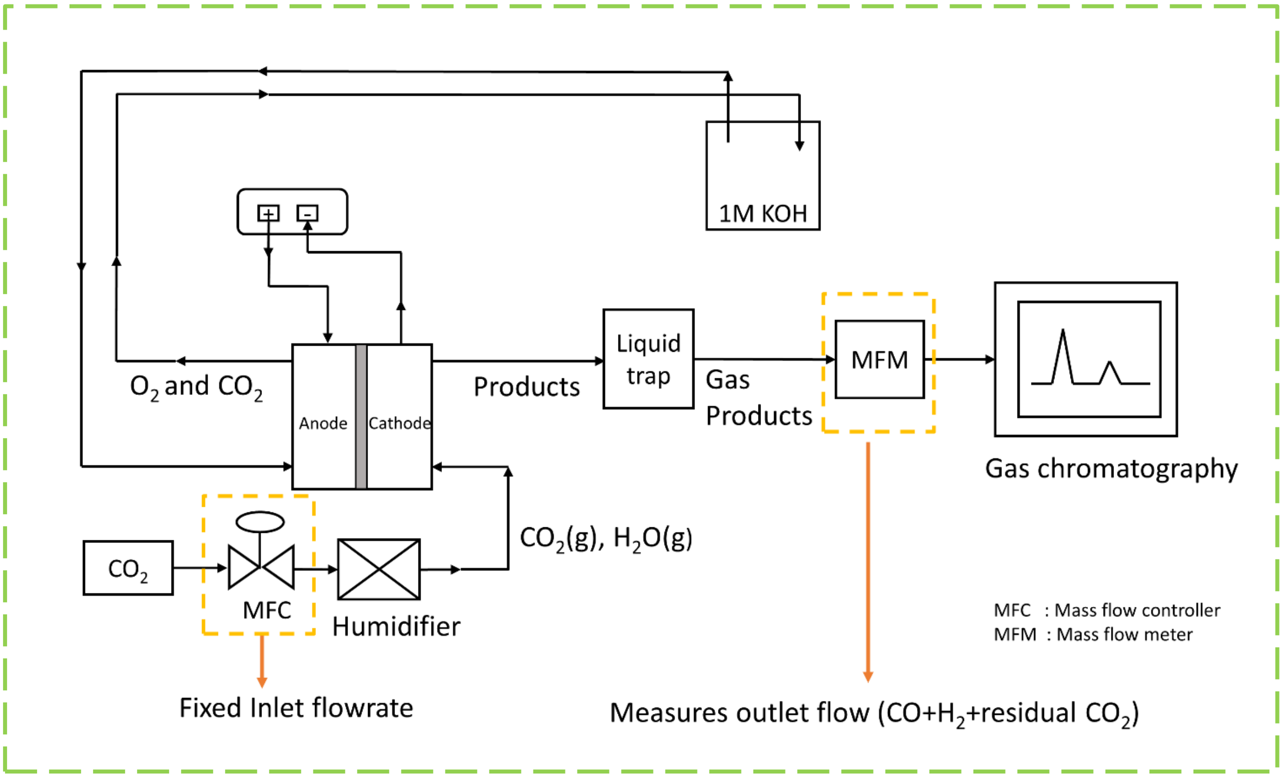

Fig. S1: Flow diagram of the experimental setup used for CO_2 electroreduction in an exchange MEA.

CO_2 was humidified by bubbling dry CO_2 into a water bath at room temperature and the relative humidity was measured using a humidity sensor. The MEA was prepared by physical compression of the electrodes and endplates using a torque wrench which were tightened to 4 Nm. This value was chosen to enhance the contact between the GDE and membrane while simultaneously ensuring that no physical damage occurred to the carbon GDE.

A series of constant current electrolysis experiments with different reactant flow rates were performed and the gaseous products from the cell were analysed using an online gas chromatography connected to the outlet of the cell equipped with two thermal conductivity detectors and a flame ionization detector. All experiments were performed for 1 hour at a current density of 200 mA/cm^2. Aliquots were collected every 5 min during the reaction resulting in a total of 12 injections in 1 hour. The concentration of gaseous products (CO and H_2) were obtained from GC and the average of 12 injections was used to calculate their faradaic efficiencies. The anolyte samples were collected after each experiments to quantify liquid products produced using HPLC measurements (Agilent Technologies). Over long enough operating periods salt formation will occur in the GDL and in the CO_2 gas channel, impacting CO_2 transport and reaction selectivity. Within the designated operating current density and testing time of 1 hr however, we did not observe any notable changes in selectivity although some salt precipitation was observed.

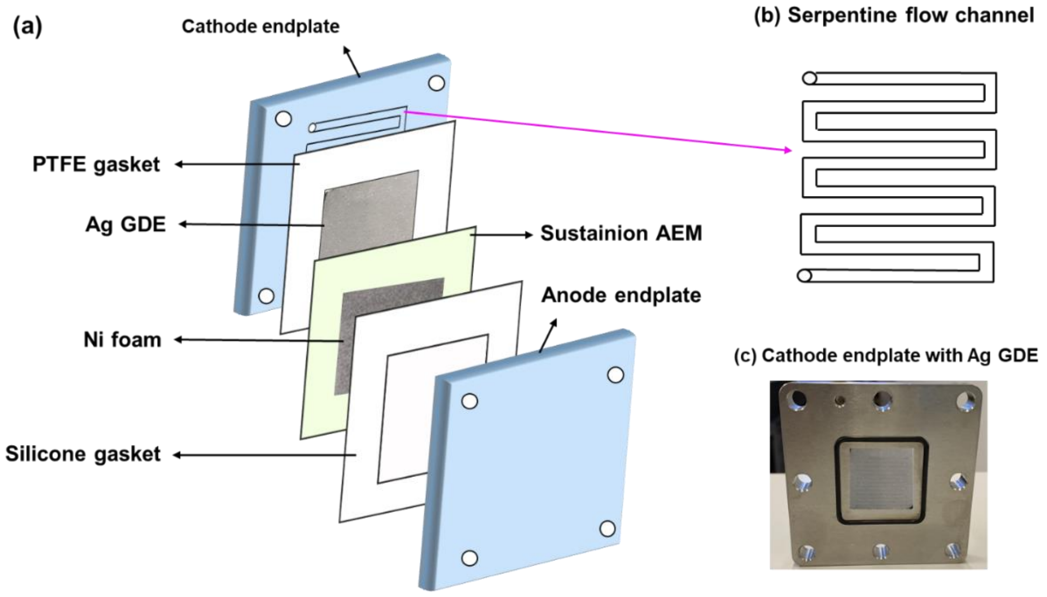

Fig. S2: (a) Schematic of the membrane electrode assembly configuration used in the experiments. (b) Serpentine flow channel with alternating turns at the endplate and (c) Dioxide materials cathode endplate with Ag GDE used in the study.

The flow rate at the outlet of the reactor was measured using a mass flow meter (Bronkhorst) in order to estimate the faradaic efficiency of products accurately. A LABVIEW program was built and connected to the mass flow meter for continuous monitoring of the outlet flowrate. The experimental

setup and the entire system design used is shown in Fig.S1. The outlet flow rate of the gas mixture (CO+H_2 +residual CO_2) from the reactor was measured ($\dot{V}_{outlet}$) using the mass flow meter and the mole fractions of CO (x_{CO}) and H_2 (x_{H_2}) were estimated from the GC injections. All the calculated values are reported in Table S2.

Faradaic efficiency calculation

To estimate the Faradaic efficiency of gaseous products, the mole fractions of CO and H_2 were estimated from GC injections. The volume fraction of gas products from GC is equal to the mole fraction for ideal gases. The mole fraction of water vapour exiting the reactor was measured using a humidity sensor and found to be 78% (x_{H2O} = 0.023). Since the sum of mole fractions is equal to 1, the mole fraction of CO_2 exiting was calculated as,

$$x_{CO2,out} = 1 - (x_{CO} + x_{H2O} + x_{H2}) \tag{S1}$$

After calculating the mole fractions of all gaseous products, the volumetric flow rate at the outlet of the reactor was measured with the MFM which was used to calculate the moles of each product.

$$n_{CO} = \frac{P\dot{V}_{outlet} \times x_{CO}}{RT} \tag{S2}$$

$$n_{H2} = \frac{P\dot{V}_{outlet} \times x_{H2}}{RT} \tag{S3}$$

$$FE_{CO} = \frac{n_{CO} \times n^{e} \times F}{I} \times 100\ \% \tag{S4}$$

Here: n_{CO} – moles/s of CO produced, n^e- number of electrons involved in CO_2RR (2 for CO), F- 96485 C/mol and I - applied current (in Amperes).

Sample calculation of FE of gas products

For an inlet flow rate of 50 sccm, the measured outlet flowrate was 39.08 ml/min which is a mixture of CO, H_2 , H_2O (g) and residual CO_2. Since the mass flow meter was calibrated for CO_2, a correction factor based on the gas conversion factors for each of the gases was used to correct the outlet flow rate. The gas conversion factor for the gas mixture outlet is given by,

$$\frac{1}{C_{mix}} = \frac{V_1}{C_1} + \frac{V_2}{C_2} + \frac{V_3}{C_3} + \frac{V_4}{C_4} \tag{S5}$$

Here C_{mix} is gas conversion factor for outlet gas mixture, V_i is the volume fraction of gas 'i'in the outlet of reactor measured from GC and C_i is gas conversion factor for gas 'i'.

Table S1: Gas conversion factors and volume fraction of gases measured from GC.

Species	Volume fraction (V_i)	Gas conversion factor
CO	0.153	C1 = 1
H_2	0.0067	C2 = 1.01
H_2O	0.023	C3 = 0.74
CO_2	1-(0.153+0.0067+0.023) = 0.817	C4 = 0.79

$$\frac{1}{C_{mix}} = \frac{0.153}{1} + \frac{0.0067}{1.01} + \frac{0.023}{0.79} + \frac{0.817}{0.74}$$

$$\Rightarrow C_{mix} = 0.773$$

$$\dot{V}_{outlet} = \frac{39.08}{0.74} \times C_{mix} = 40.82\ ml/min$$

$$FE_{CO} = \frac{2 \times 96485\ Cmol^{-1} \left(\frac{0.153 \times \frac{40.82}{60} \times 10^{-6}\ m^3 s^{-1}}{8.314 \frac{J}{Kmol} \times 273K} \right) \times 101325\ Pa}{1.25\ A} \times 100 = 71.7\ \%$$

$$FE_{H2} = \frac{2 \times 96485\ Cmol^{-1} \left(\frac{0.007 \times \frac{40.82}{60} \times 10^{-6}\ m^3 s^{-1}}{8.314 \frac{J}{Kmol} \times 273K} \right) \times 101325\ Pa}{1.25\ A} \times 100 = 3.15\ \%$$

Carbon balance at the cathode

The following equations were then used to calculate the CO_2 consumption with OH^- ions and make a carbon balance on the cathode side.

$$\dot{V}_{CO_2\ to\ CO} = x_{CO} \times \dot{V}_{outlet} \quad \text{(S6)}$$

$$\dot{V}_{H_2} = x_{H_2} \times \dot{V}_{outlet} \quad \text{(S7)}$$

$$\dot{V}_{residual\ CO_2} = \dot{V}_{outlet} - \left(\dot{V}_{CO_2\ to\ CO} + \dot{V}_{H_2}\right) \quad \text{(S8)}$$

$$\dot{V}_{CO_2\ to\ HCOO^-} = ((1 - x_{CO} - x_{H2}) \times \frac{j\times A}{n^e \times F} mol\ s^{-1} \times 22.4\ L\ mol^{-1} \times 60\ s \times 1000\ )\ ml/min \quad \text{(S9)}$$

$$\dot{V}_{OH^-} = \dot{V}_{inlet} - (\dot{V}_{residual\ CO_2} + \dot{V}_{CO_2\ to\ CO} + \dot{V}_{CO_2\ to\ HCOO^-}) \quad \text{(S10)}$$

$$CO_2\ utilization\ = \frac{(\dot{V}_{CO_2\ to\ CO} + \dot{V}_{CO_2\ to\ HCOO^-})}{\dot{V}_{inlet}} \times 100\% \quad \text{(S11)}$$

$$j_{loss} = \frac{n^e \times \dot{V}_{CO2\ to\ OH-} \times F}{A} \quad \text{(S12)}$$

Table S2 shows the carbon balance performed on the cathode side from which the fraction of CO_2 reacting with OH^- ions was calculated. Here, measuring the flowrate of gas products at the outlet of the reactor is an important factor in the estimation of FE of gas products and CO_2 losses.

We observed that the sum of FE of CO and H_2 did not add upto 100 % which is possibly due to the formation of some liquid products. To determine this, we collected the anolyte (1M KOH) samples post electrolysis and conducted high performance liquid chromatography (HPLC) analysis. Formate ($HCOO^-$) was the only product detected showing that formate ions produced at the cathode migrates to the anolyte through the AEM. The sum of FE of CO, H_2 and formate reached 96-97.5 % for most of the studied inlet flow rates and we suspect that the remaining formate ions possibly oxidized to CO_2 at the anode as reported previously. After this confirmation, we calculated the FE of formate produced as 100- (FE_{CO}+FE_{H2}) in order to make a carbon balance at the cathode side with the assumption that no non-faradaic reactions take place. The amount of CO_2 lost to OH- ions was then calculated for all the studied inlet flowrates.

Table S2: CO_2 losses, utilization rate and modified inlet flow rates and j_{loss} used in the model at an applied current density of 200 mA/cm^2.

Inlet flow rate (sccm)	$\dot{V}_{CO_2\ to\ CO}$ (ml/min)	$\dot{V}_{CO_2\ to\ HCOO-}$ (ml/min)	$\dot{V}_{residual\ CO_2}$ (ml/min)	$\dot{V}_{OH^-}$ (ml/min)	CO_2 utilization (%)	Case B- Modified inlet flow rate for the model (ml/min)	Case C- j_{loss} due to CO_2 lost to OH^- ions (mA/cm^2)
10	3.13	1.95	0.69	4.41	50.8	5.59	106.4
12.5	3.72	2.55	0.81	5.55	50.1	6.95	127.5
15	4.15	3.29	1.67	6.19	49.6	8.81	142.2
17.5	4.41	3.63	2.58	6.78	45.9	10.72	155.7
20	5.49	2.65	4.83	7.35	40.7	12.65	168.9
25	5.65	2.2	10.61	7.41	31.4	17.59	170.3
30	5.93	2.14	14.40	8.01	26.9	21.99	184
40	6.17	2.02	23.80	8.78	20.5	31.22	201.7
50	6.18	2.24	33.25	9.31	16.8	40.69	214.1

($\dot{V}_{CO_2\ to\ CO}$: consumed CO_2 flow rate which is electrochemically converted to gas product (CO); $\dot{V}_{residual\ CO_2}$: unreacted CO_2 flowrate in the gas outlet; $\dot{V}_{OH^-}$: consumed CO_2 flowrate via the reaction with OH^-.)

Modified current density used in the model

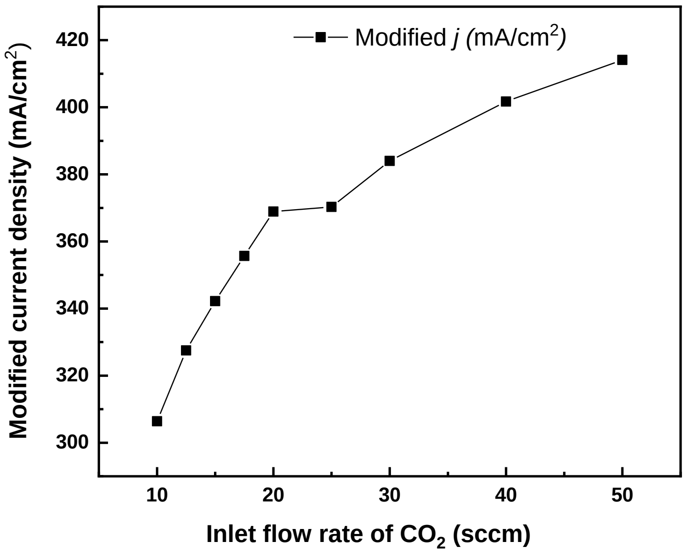

Fig S3. Modified current density used in the model after taking into account of the fraction of CO_2 lost to hydroxide ions from carbon balance. This represents the non-Faradaic consumption of CO_2 in the model.

Outlet flow rate and Faradaic efficiency measurement during electrolysis

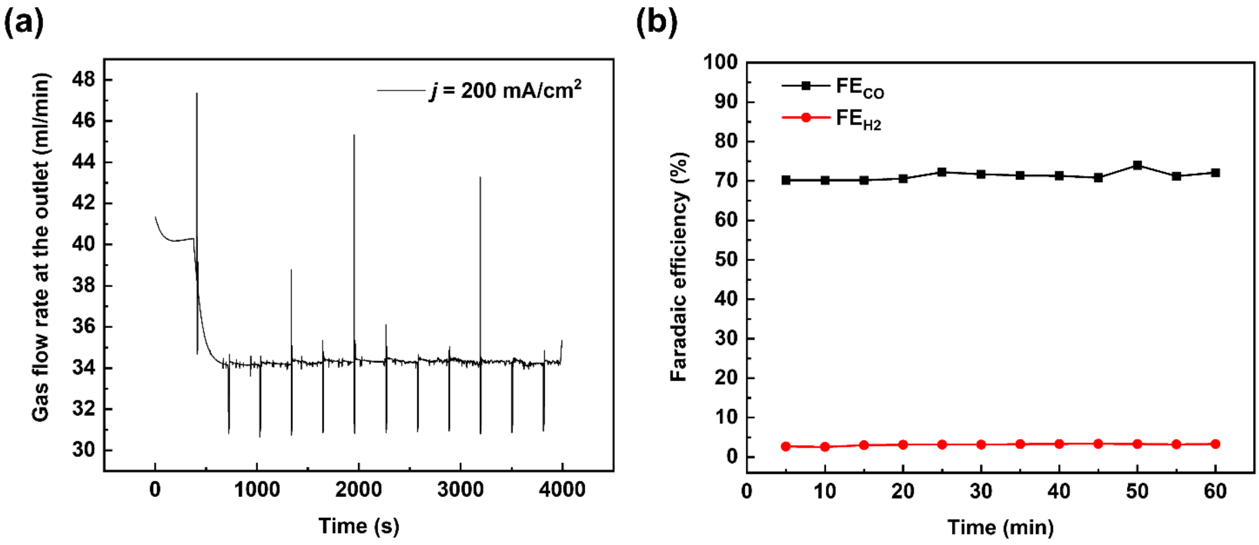

Fig S4: (a) Measured outlet flowrate during electrolysis at 200 mA/cm^2 for an inlet flow rate of 50 sccm. The dip in the peaks occurring every 5 min are due to the periodic GC injections. (b) Faradaic efficiency of CO and H_2 measured from the GC injections.

Flow rate of humidified CO_2

The flowrate of humidified CO_2 entering the reactor varies slightly for each CO_2 flow rate. So we measured the flowrate of humidified CO_2 to make the carbon balance accurately, specifically in the determination of the amount of CO_2 reacting with OH^- ions since it depends on the inlet flowrate as shown in equation S10.

Table S3: Flowrate of humidified CO_2 used in the experiments

Inlet flowrate of CO_2 (sccm) measured from MFC	Inlet flowrate of humidified CO_2 (sccm)
10	10.1
12.5	12.6
15	15.1
17.5	17.6
20	20.2
25	25.3
30	30.5
40	40.8
50	51.0

Humidity measurements

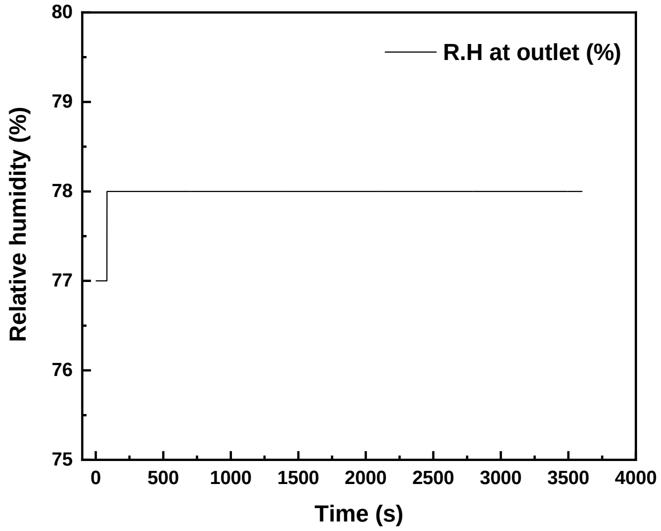

Fig. S5. Relative humidity measured at the exit of the reactor during electrolysis at 200 mA/cm^2 for an inlet flow rate of 50 sccm.

The relative humidity (R.H) at the outlet of the reactor was measured during electrolysis and it remained constant throughout 1 hour electrolysis. It did not vary significantly for different inlet flow rates. The measured R.H for an inlet flow rate of 50 sccm of CO_2 is shown in Fig S5. As shown here, R.H at outlet was 78 % corresponding to a mole fraction of 0.023 for H_2O which was used in FE calculation.

Table S4: Liquid products analysis using HPLC

Inlet flow rate (sccm)	$HCOO^-$ detected from HPLC (in ppm)	FE of $HCOO^-$ (%)	Predicted FE of $HCOO^-$ (100-FE_{CO}-FE_{H2} %)	Missing FE (%)
10	2228.05	21.23	25.16	3.92
12.5	2953.5	28.14	31.94	3.79
15	2546.5	24.28	33.41	9.13
17.5	2372.9	22.61	32.2	9.59
20	2955.1	28.16	30.54	2.38
25	2681.1	25.54	29.66	4.11
30	2164.9	20.63	22.98	2.34
40	1976.1	18.83	22.71	3.87
50	1582.1	15.07	24.6	9.73

Faradaic efficiency of Formate from HPLC

Sample calculation: The formate ions present in the anolyte were detected from HPLC measurements and the faradaic efficiency was calculated using the following equation. Here a sample calculation using 40 sccm as inlet flow rate is shown.

$$[HCOO^-]\ detected = 1976.1\ ppm = 1.976\ gL^{-1}$$

$$Molecular\ weight\ of\ HCOO^- = 45\ gmol^{-1}$$

$$Volume\ of\ anolyte\ used = 0.1\ L$$

$$Moles\ of\ HCOO^- = \frac{1.976 gL^{-1}}{45\ gmol^{-1}} \times 0.1\ L = 4.39 \times 10^{-3}\ \ moles$$

$$FE_{HCOO^-} = \frac{Moles\ of\ HCOO^- \times n^e \times F}{I \times t} \times 100\ \% = \frac{4.39 \times 10^{-3} \times 2 \times 96485}{1.25\ A \times 3600\ s} = 18.83\ \%$$

$$Sum\ of\ FE\ of\ products = FE_{CO} + FE_{HCOO^-} + FE_{H2} = (71.5 + 18.83 + 5.7)\% = 96.03\ \%$$

Table S5: Error associated with outlet gas flow rate and translation to measured gaseous FE's

Inlet flow rate (sccm)	$Outlet\ flow\ rate$ $\dot{V}_{outlet}$ (ml/min)	FE_{CO} (%)	FE_{H2} (%)	$Error\ in\ FE_{CO}$(%)	$Error\ in\ FE_{H2}$ (%)
10	7.18 ±1	35.98	38.86	4.93	5.3
12.5	6.88 ±1	42.76	25.3	6.31	3.7
15	7.43 ±1	54.2	12.35	7.72	1.78
17.5	9.23 ±1	60.4	7.2	6.46	0.74
20	11.14 ±1	62.4	7.06	5.78	0.75
25	16.27 ±1	64.9	9.3	3.1	0.39
30	21.38 ±1	69.9	7.1	3.2	0.38
40	31.21 ±1	71.5	5.79	2.28	0.28
50	40.62 ±1	73.05	3.05	1.77	0.12

Model description

A 3D geometry of the cathode compartment (5cm^2 area) comprising of the serpentine flow channel with a series of alternating 180^0 turns and 12 ribs with same length (2.25 cm), width (1 mm) and depth (1 mm) was modeled in COMSOL Multiphysics. A carbon GDL of dimensions - 2.5cm x 2.5cm x 325µm was placed in contact with the gas flow channel. The numerical simulations were performed using a MUMPS general solver with a relative tolerance of 0.001 to calculate the CO_2 concentration gradient in the gas channels and catalyst surface. For Case A and Case B, a current density of 200 mA/cm^2 was applied at the catalyst surface. For Case C, the modified current density for each of the inlet flow rate was used to estimate the concentration gradient.

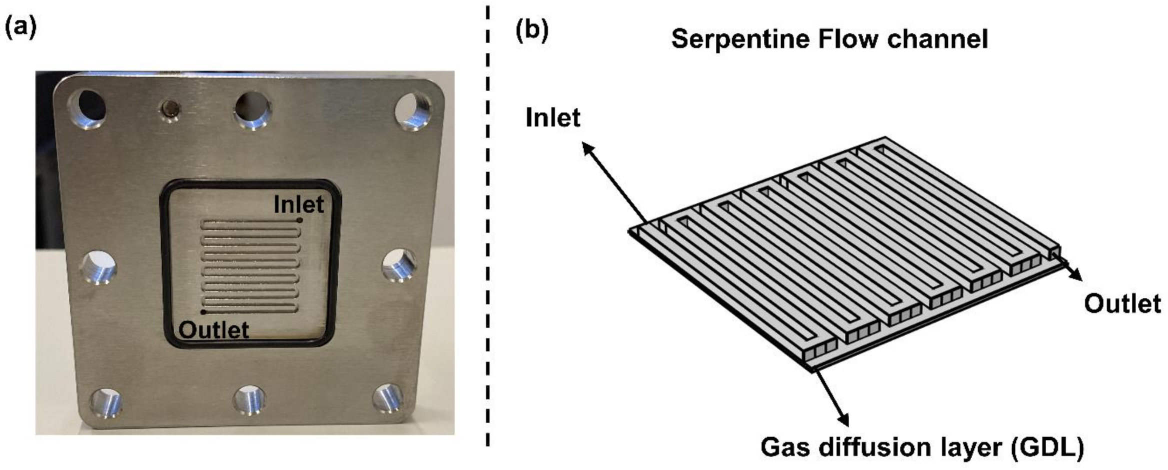

Fig. S6: (a) 5 cm^2 cathode flow plate used in the experiments comprising the serpentine flow channel and (b) 3D model of the cathode compartment of MEA modelled in COMSOL Multiphysics.

Electrochemical reduction of CO_2 to CO was modelled and the competing hydrogen evolution reaction was not taken into account. The electrochemical reduction reaction occurring at the cathode is a 2 e$^-$ reduction reaction:

$$CO2\,RR:\; CO_2 + H_2O + 2e^- \rightarrow CO + 2OH^- \quad (3.1)$$

All parameters used in the model were taken from the experimental setup. The following assumptions were made in the model:

i) The system operates at steady-state conditions
ii) Carbon GDL is assumed to be isotropic with constant porosity and permeability since the in-plane diffusion is higher than the through plane diffusion
iii) Both diffusion and convection from the channel to the catalyst surface are taken into account
iv) A uniform current distribution is assumed on the catalyst surface
v) Isothermal at 298 K with no thermal diffusion gradients

Brinkmann equation and mixture diffusion model

The fluid flow in the serpentine channels was modelled using the Brinkmann equations which is a modified form of the Navier stokes equation for porous media flows. A no-slip boundary condition was imposed on the channel walls. A slip condition was used at the channel-GDL interface since the normal component of velocity is zero at this interface. Single phase compressible flow was assumed. An inlet boundary condition was given by a normal inflow velocity defined by the flowrate ($\dot{V}_{in}$) over inlet cross sectional area of the channel (Dirichlet boundary condition). $\dot{V}_{in}$ varied from 10 to 50 sccm which was used in the experiments. A zero pressure boundary condition was imposed at the outlet (Neumann boundary condition) with the suppression of backflow.

The Brinkman equation solves for the velocity and pressure distribution in the GDL. It was coupled with the mixture diffusion model taking into account of the diffusion and convection through the GDL. All parameters used in the model can be found in Table S6. For the meshing, a free tetrahedral mesh with a fine mesh size was used for the channels and a swept mesh was used for the GDL (98023 domain elements, 24196 domain elements and 2894 edge elements) resulting in a run time for 45 minutes for every simulation. The velocity and pressure field in the gas channels were solved using:

$$\rho(\mathbf{u}\cdot\boldsymbol{\nabla})\mathbf{u} = \boldsymbol{\nabla}\cdot\left[-p\boldsymbol{I} + \mu(\nabla\mathbf{u} + (\nabla\mathbf{u})^{\mathrm{T}}) - \frac{2}{3}\,\mu(\nabla\cdot\mathbf{u})\mathbf{I}\right] + \mathrm{F} \tag{S13}$$

$$\nabla\cdot(\rho\mathbf{u}) = 0 \tag{S14}$$

In the GDL, the velocity and pressure was calculated using:

$$\frac{1}{\epsilon_p}\rho(\mathbf{u}\cdot\nabla)\mathbf{u}\frac{1}{\epsilon_p} = \nabla\cdot\left[-p\mathbf{I} + \mu\frac{1}{\epsilon_p}(\nabla\mathbf{u} + (\nabla\mathbf{u}^{\mathrm{T}}) - \frac{2}{3}\mu\frac{1}{\epsilon_p}(\nabla\cdot\mathbf{u})\mathbf{I}\right] - \left(\mu\kappa^{-1} + \frac{Q_m}{\epsilon_p^2}\right)\mathbf{u} + \mathrm{F} \tag{S15}$$

$$\nabla\cdot(\rho\mathbf{u}) = Q_m \tag{S16}$$

In these equations,

ρ is the density of the fluid, μ is the dynamic viscosity of the fluid, p is the pressure, $\mathbf{u}$ is the velocity, F is the force term, κ is the permeability of the GDE, ϵ_p is the porosity of the GDE and Q_m is the mass source.

Mixture diffusion model

To solve for the species transport in the system, a mixture diffusion model was used. Relative humidity in the inlet stream was ignored since the humidity measured experimentally at the inlet remained constant at 75%. So, we accounted for only 2 species which are CO_2 and CO. The molar flux of the species were calculated using the following equations:

$$\nabla\cdot j_i + \rho(\mathbf{u}\cdot\nabla)\omega_i = R_i \tag{S17}$$

$$N_j = j_i + \rho \mathbf{u} \omega_{\boldsymbol{i}} \quad \text{(S18)}$$

$$\boldsymbol{j}_i = -(\rho D_i^m \nabla \omega_i + \rho \omega_i D_i^m \frac{\nabla M_n}{M_n}) \quad \text{(S19)}$$

$$R_i = \frac{v_i i_v}{nF} \quad \text{(S20)}$$

Here:

N is the total flux vector of species i, R_i is the reaction rate for species i, **u** is the fluid velocity, $\mathbf{j}_i$ is the relative mass flux due to molecular diffusion of species i, ω_i is the mass fraction of species i, i_v is the volumetric current density, F- Faraday's constant. Here, equation S18 represents the convection-diffusion equation with the first term representing diffusion and second term representing convection the magnitude of which depends on the inlet velocity 'u'.

Mesh independence study

A mesh independence study was performed to ensure that the right mesh size was chosen. An element size of 0.5 mm was chosen for the free tetrahedral mesh that generated a total of 98023 domain elements. Fig. S7 shows the mesh independence study performed to estimate the average CO_2 concentration at the catalyst surface for an inlet flowrate of 50 sccm and 200 mA/cm^2.

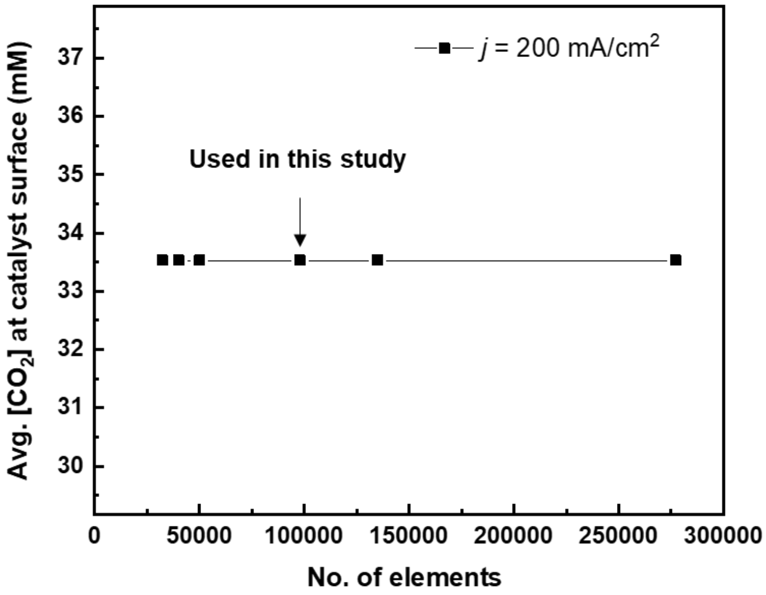

Fig. S7. A mesh independence study performed with different mesh element sizes at an inlet flowrate of 50 sccm and a current density of 200 mA/cm^2. The average CO_2 concentration at the catalyst surface was calculated and did not vary within the shown number of domain elements.

Simulation results

The simulation results of CO_2 concentration in the gas channels and at the catalyst surface are shown in Fig.S8 for an inlet flow rate of 10 sccm. Here the CO_2 losses to OH^- ions are ignored (Case A).

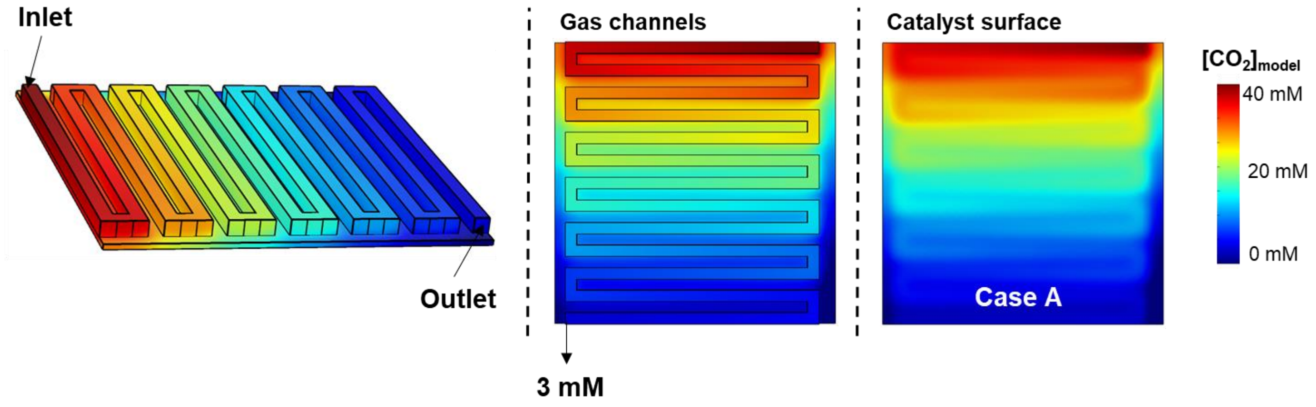

Fig. S8. CO_2 concentration at the catalyst surface for an inlet flow rate of 10 sccm and current density of 200 mA/cm^2 without accounting for the fraction of CO_2 reacting with hydroxide ions.

CO_2 concentration at different inlet flow rates

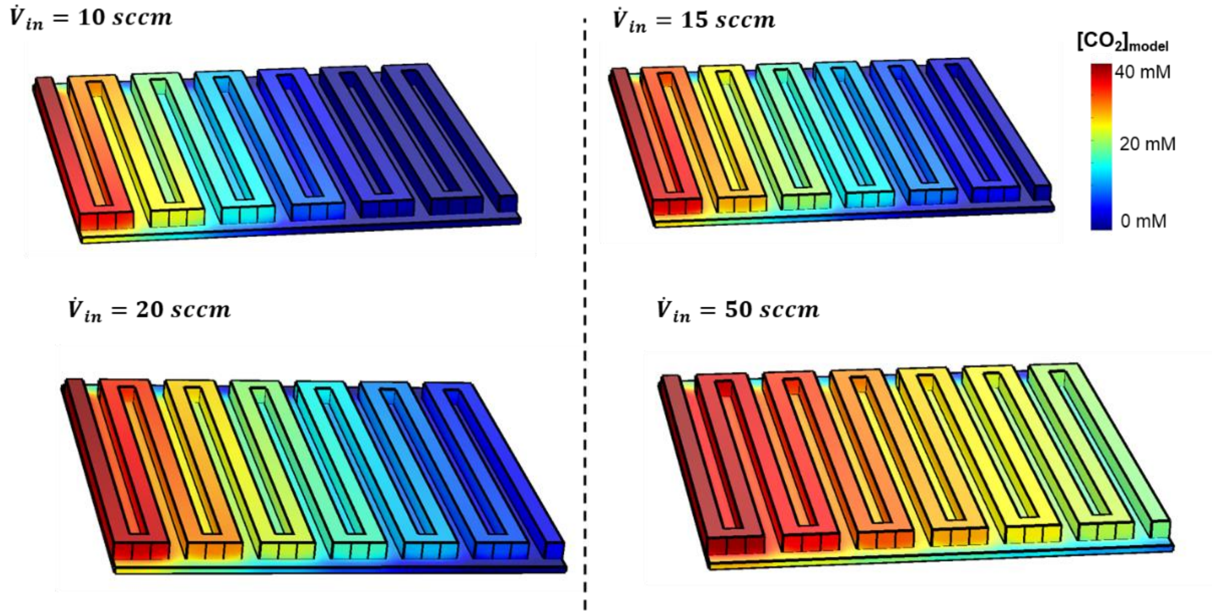

Fig. S9. CO_2 concentration at the cathode for different inlet flow rates using a modified current density approach (Case C). Here, the CO_2 losses to OH^- occurring homogenously throughout the catalyst surface are considered.

Average CO_2 concentration at the catalyst surface

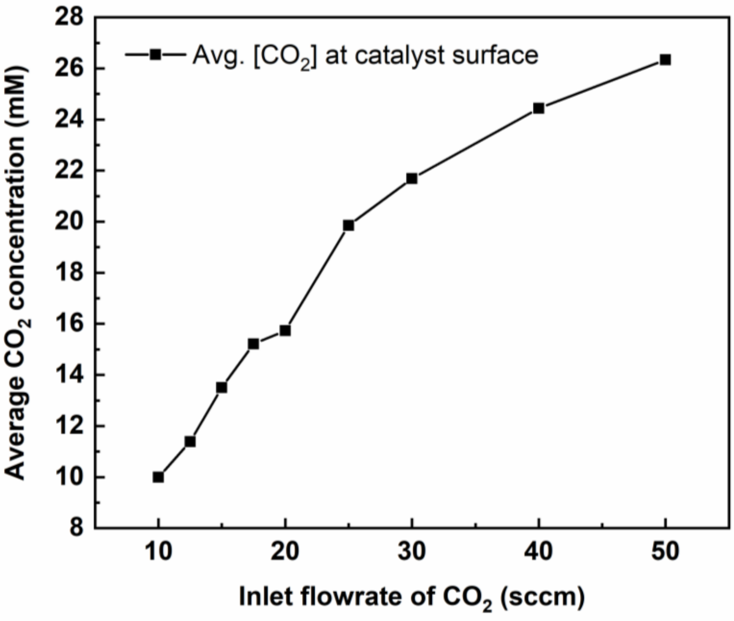

Fig. S10. The average CO_2 concentration at the catalyst surface for the studied inlet flow rates calculated from the model (Case C).

Cumulative distribution plots of CO_2 concentration at the catalyst surface

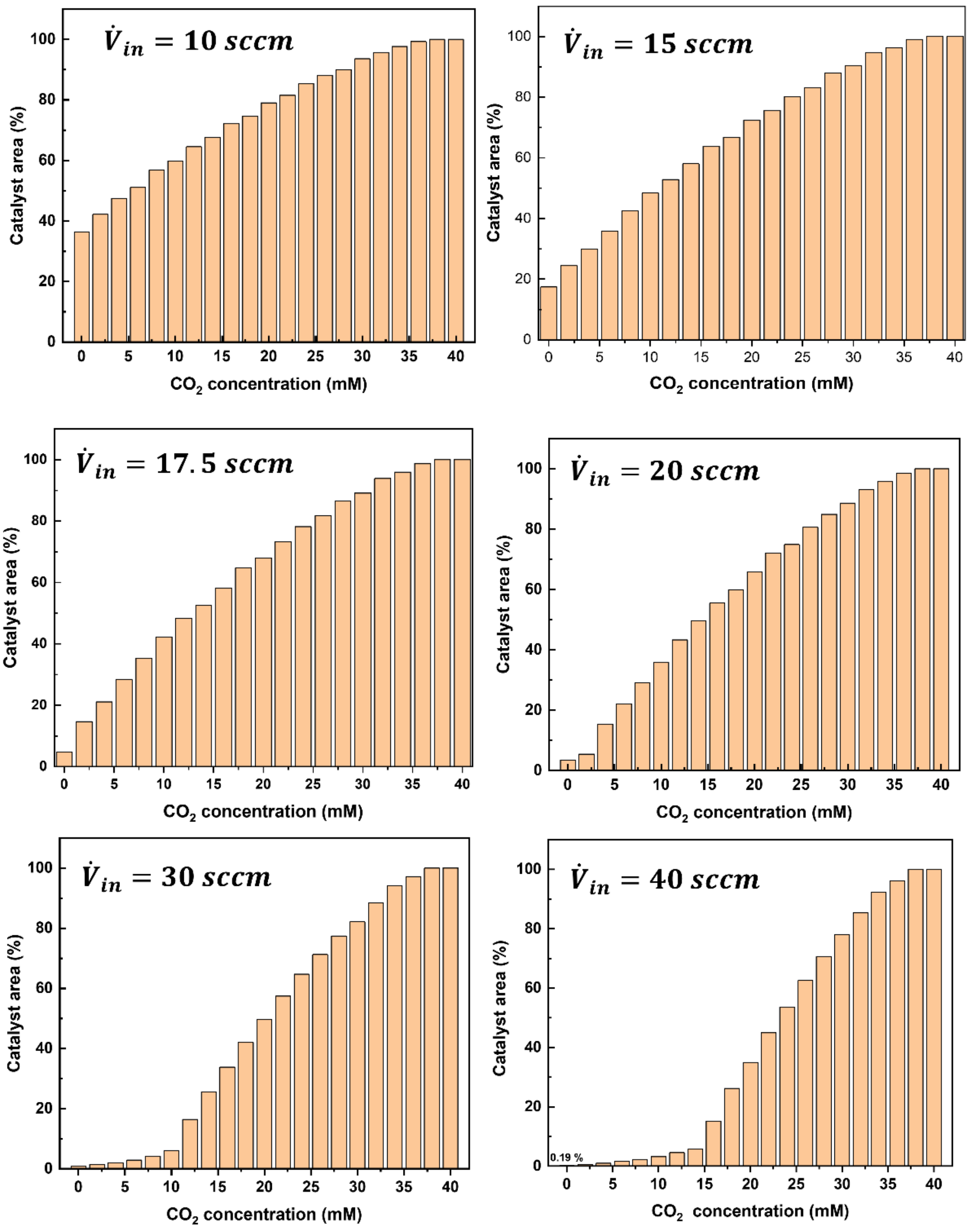

Fig. S11: Cumulative distribution plots of CO_2 concentration at the catalyst surface for various inlet flowrates using a modified current density approach (Case C).

Catalyst area having [CO_2]= 0 at different inlet flowrates

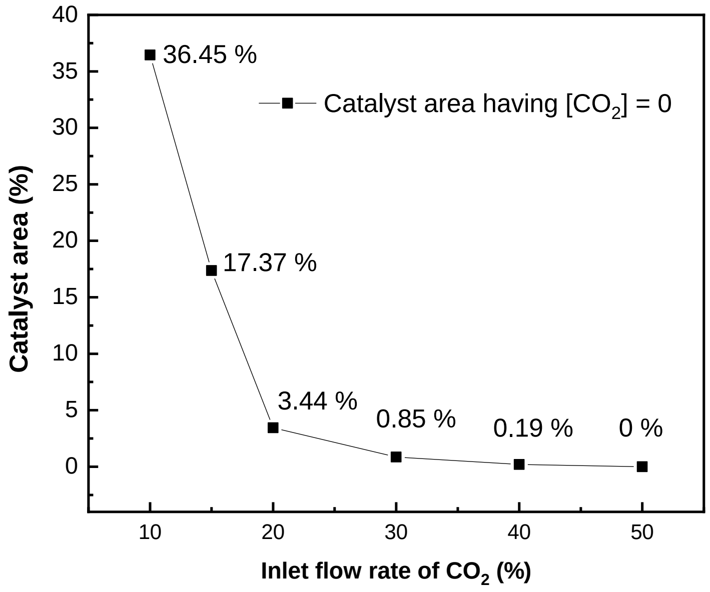

Fig. S12: Percentage of catalyst surface area having [CO_2] = 0 for various inlet flowrates using a modified current density approach (Case C).

Other Figures and Tables

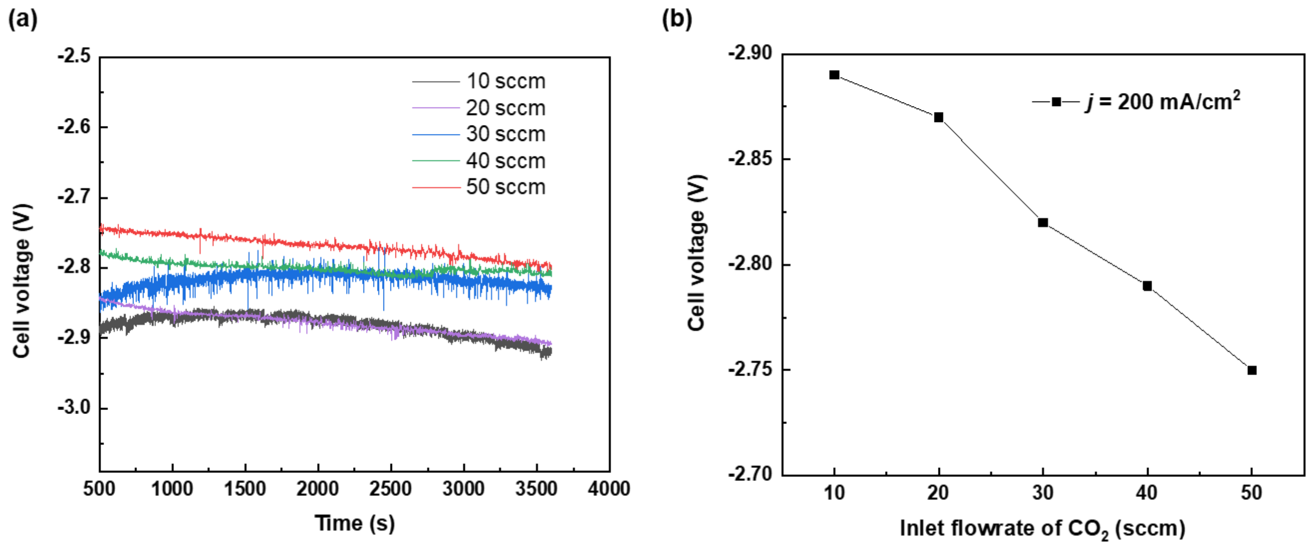

Fig. S13. (a) Variation of cell voltage with time during CO_2 electrolysis for various inlet CO_2 flow rates. **(b)** Cell voltage for various inlet CO_2 flowrates at a current density of 200 mA/cm^2.

Material characterization

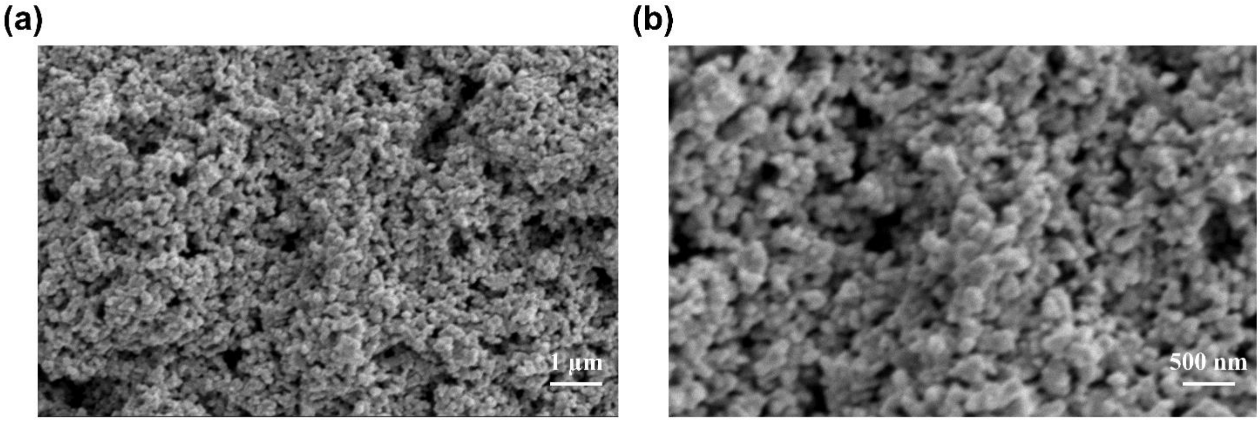

Fig.S14. SEM images of Ag catalysts coated on microporous layers of Sigracet 38 BC gas diffusion layers.

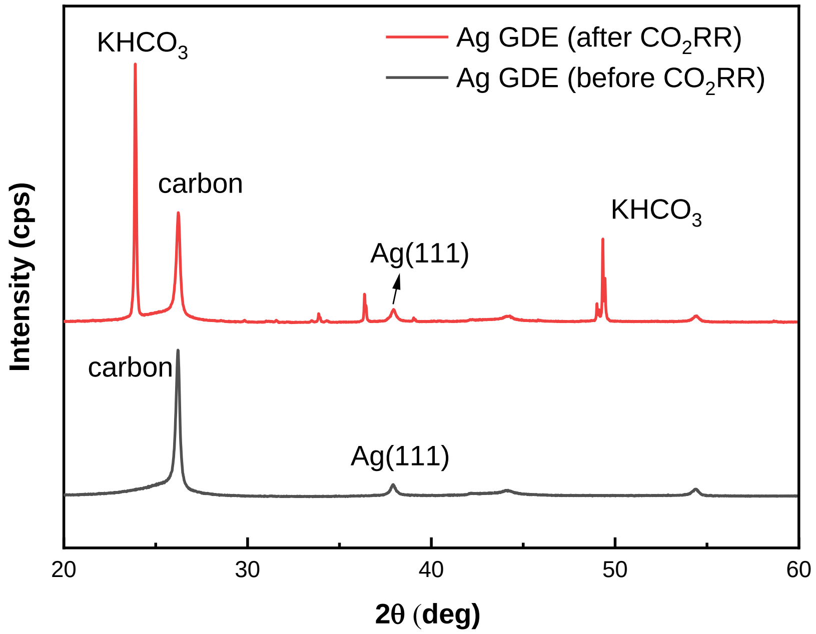

Fig. S15: XRD pattern of Ag GDE before and after CO_2 electrolysis showing the presence of Ag (111) facet. Potassium bicarbonate salt peaks are visible due to salt precipitation at the cathode.

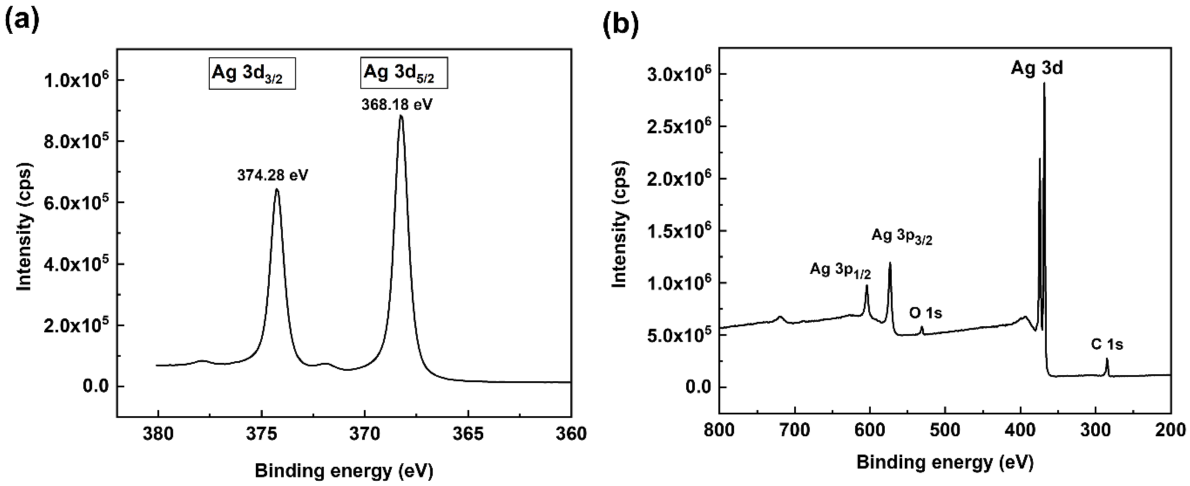

Fig. S16: XPS analysis of Ag catalysts coated on microporous layers of Sigracet 38 BC gas diffusion layers.

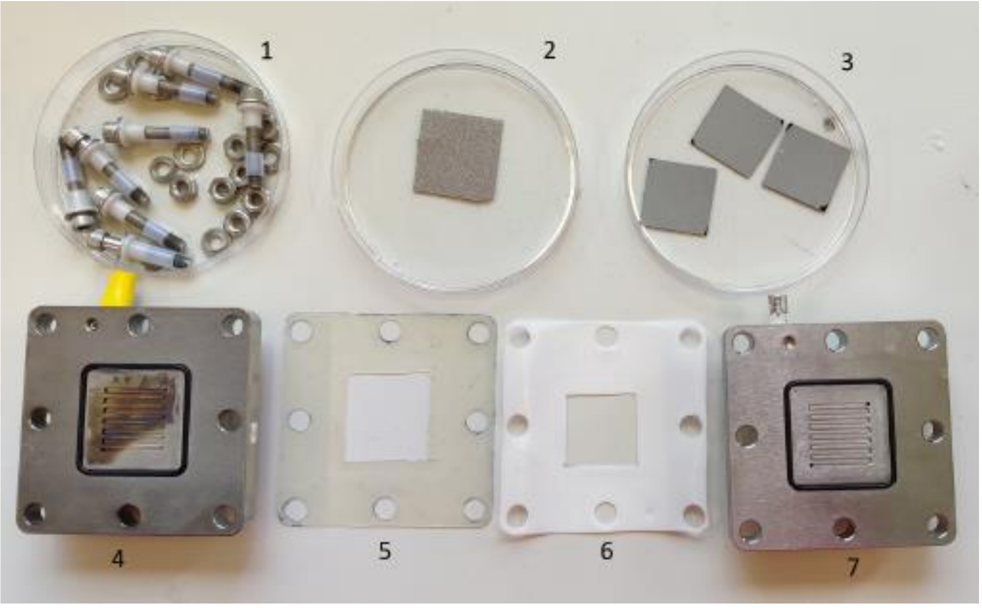

Fig. S17: Components used in the MEA setup. 1: Nuts and bolts, 2: Ni porous medium , 3: GDE with Ag sputtered catalyst layer, 4: Titanium anode endplate, 5: Silicone gasket for anode, 6: PTFE gasket for cathode, 7: Stainless steel cathode endplate.

Table S6 : Parameters used in the 3D mass transport and fluid flow model

Parameter	Symbol	Value	Units	Reference
Temperature	T	298	K	This work
Reference pressure	P	1	atm	This work
Diffusivity of CO into CO_2	D_{CO2-CO}	1.52×10^{-5}	$m^2 s^{-1}$	[1]
Porosity of GDL	eps_gdl	0.8	$-$	[2]
Permeability of GDL	kappa_gdl	7×10^{-12}	m^2	[2]
Inlet flowrate	Q_{sccm}	10-50	sccm	This work
Applied current density	i_{loc}	-2000	Am^{-2}	This work

Table S7: Electrochemical and homogenous reactions occurring at the cathode of the electrolyzer.

CO_2RR to CO	$CO_2 + H_2O + 2e^- \rightarrow CO + 2OH^-$	(S12)
HER	$2H_2O + 2e^- \rightarrow H_2 + 2OH^-$	(S13)
CO_2RR to $HCOO^-$	$CO_2 + H_2O + 2e^- \rightarrow HCOO^- + OH^-$	(S14)
HCO^{3-} formation	$CO_2 + OH^- \leftrightarrow HCO_3^-$	(S15)
CO_3^{2-} formation	$HCO_3^- + OH^- \leftrightarrow CO_3{}^{2-}$	(S16)

References

[1] E. L. Cussler, *Diffusion, mass transfer in fluid systems*, Cambridge University Press, Cambridge Cambridgeshire ; New York, 1984.

[2] R. Schweiss, C. Meiser, T. Damjanovic, I. Galbiati and N. Haak, *SIGRACET® Gas Diffusion Layers for PEM Fuel Cells, Electrolyzers and Batteries (White Paper)*, SGL CARBON GmbH, 2016.

[3] COMSOL Inc, *CFD Module User's Guide*, 2017.

[4] L.-C. Weng, A. T. Bell and A. Z. Weber, *Energy Environ. Sci.*, 2019, **12**, 1950–1968.
